# Small Object Detection and Tracking: A Comprehensive Review

**DOI:** 10.3390/s23156887

**Published:** 2023-08-03

**Authors:** Behzad Mirzaei, Hossein Nezamabadi-pour, Amir Raoof, Reza Derakhshani

**Affiliations:** 1 Intelligent Data Processing Laboratory (IDPL), Department of Electrical Engineering, Shahid Bahonar University of Kerman, Kerman 76169-13439, Iran; 2 Department of Earth Sciences, Utrecht University, 3584CB Utrecht, The Netherlands; 3 Department of Geology, Shahid Bahonar University of Kerman, Kerman 76169-13439, Iran

**Keywords:** small object, detection, tracking, computer vision, survey

## Abstract

Object detection and tracking are vital in computer vision and visual surveillance, allowing for the detection, recognition, and subsequent tracking of objects within images or video sequences. These tasks underpin surveillance systems, facilitating automatic video annotation, identification of significant events, and detection of abnormal activities. However, detecting and tracking small objects introduce significant challenges within computer vision due to their subtle appearance and limited distinguishing features, which results in a scarcity of crucial information. This deficit complicates the tracking process, often leading to diminished efficiency and accuracy. To shed light on the intricacies of small object detection and tracking, we undertook a comprehensive review of the existing methods in this area, categorizing them from various perspectives. We also presented an overview of available datasets specifically curated for small object detection and tracking, aiming to inform and benefit future research in this domain. We further delineated the most widely used evaluation metrics for assessing the performance of small object detection and tracking techniques. Finally, we examined the present challenges within this field and discussed prospective future trends. By tackling these issues and leveraging upcoming trends, we aim to push forward the boundaries in small object detection and tracking, thereby augmenting the functionality of surveillance systems and broadening their real-world applicability.

## 1. Introduction

Detecting and tracking moving objects in various forms of visual media is a critical aspect of numerous applications, from video surveillance [1] to intelligent traffic management [2] and digital city infrastructure [3]. The platforms implementing these tasks range from closed-circuit televisions (CCTVs) [4] and aircraft [5,6] and unmanned aerial vehicles (UAVs) [7] to the transport of nano-particles in soil for environmental protection [8].

Object detection involves identifying a target object within a single frame or image, whereas object tracking focuses on estimating or predicting the object’s position throughout the video sequence, given its initial location [8,9,10]. These capabilities are essential in a wide array of computer vision tasks, including surveillance, autonomous navigation for vehicles and robots, and more. Object tracking typically occurs in two distinct contexts. Single object tracking (SOT) is focused on a singular target throughout the video, whereas multiple object tracking (MOT) or multiple target tracking (MTT) involves keeping track of numerous objects simultaneously [11]. For instance, the military may use tracking for national surveillance, including border patrols and base perimeter security, or for improving missile guidance systems. In the sporting domain, systems like Hawk-eye monitor ball movement, aiding line judges and player strategy development.

Detecting and tracking objects in infrared (IR) images is another critical application where the target might vary in size or approach at different velocities [12]. The tasks involved in detection and tracking largely depend on the granularity of information the user seeks to extract [13]. Tracking can be quite intricate due to numerous factors. Challenges arise when objects move rapidly, experience occlusion, or become invisible. Difficulties also emerge in the presence of noise, non-rigid objects, rotation, scale changes, and moving cameras. Despite notable advancements, these complexities persist, especially when tracking small objects from a considerable distance [14].

**Figure 1:**
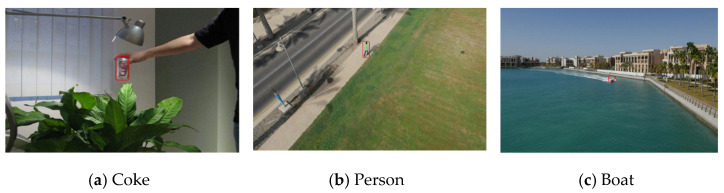
Some representative instances of small objects.

A small object is one that appears tiny within the video frame, like a distant paraglider or soccer ball, often due to being tracked from afar. These objects, common in applications such as UAVs, remote sensing, and ball sports, may exhibit poor appearance attributes and are challenging to detect and track due to their size. They can often be mistaken for noise due to their minuscule size, negatively impacting tracking accuracy. The term “small objects” is commonly defined in two ways. First, it may refer to objects that are physically smaller in the real world. Alternatively, as per the MS-COCO [15] metric evaluation, small objects are those with an area of 32 × 32 pixels or less, a threshold generally accepted for datasets involving common objects. Small object detection and tracking thus represent a specialized facet of object detection and tracking, necessitating distinct techniques for handling digital images and videos. For instance, aerial images often require advanced methods due to the inherent nature of small objects [16]. Figure 1 provides several examples of small objects.

Previous research primarily focused on the detection and tracking of larger objects within scenes, with lesser emphasis on smaller targets. This revealed a necessity for more nuanced and precise algorithms to address small object detection and tracking. Despite the prevalent usage and inherent challenges associated with small object detection and tracking, a comprehensive review focusing specifically on this subject has been noticeably absent. As such, the main purpose of this study was to thoroughly review the existing methods and provide a structured taxonomy.

The primary findings of this research can be outlined as follows:

- A detailed review of methods for detecting and tracking small objects, thereby addressing a significant research gap. To our knowledge, such a comprehensive review has not been previously conducted. The proposed taxonomies aim to provide researchers with a broader and more profound understanding of small object detection and tracking.
- Classification of existing methods into five categories: filter-based methods, search-based methods, background detection-based methods, classical computer-vision-based methods, and deep-learning-based methods.
- Segregation of public datasets used for small detection and object tracking into two categories: spectrum-based video datasets and source position-based video datasets. The datasets are introduced, and the most commonly employed evaluation metrics for detecting and tracking small objects are presented.
- A discussion on the primary challenges in this field, coupled with an analysis of the strengths and weaknesses of methods within each category. We also highlight potential future research trends.

In the ensuing sections of this paper, we propose a comprehensive taxonomy for small object detection and tracking methods. Section 2 undertakes a review of existing methods from various perspectives. Section 3 provides details on the types of public datasets used for small object detection and tracking. Section 4 presents common evaluation metrics for the detection and tracking phases. In Section 5, we delve into the current challenges in the field and propose potential future trends. Section 6 concludes the paper.

Our goal in reviewing existing methods, addressing challenges, and suggesting future directions is to advance the field of small object detection and tracking. By doing so, we hope to facilitate improvements in various computer vision applications such as surveillance systems, autonomous navigation, and object tracking in dynamic environments.

## 2. Taxonomy and Review the Small Object Detection and Tracking Methods

Although many different approaches have been suggested in the field of detection and tracking of large objects, these methods are limited for detection and tracking of small objects which can be classified into two main groups such as unified track-and-detection methods and track-by-detection methods.

Unified track-and-detection methods are grouped into two categories as filter-based methods and search-based methods. Also, track-by-detection methods are divided as three other categories, including background detection-based methods, classical computer-vision-based methods, and deep-learning-based methods. This section will provide an overview of these methods.

### 2.1. Unified Track-and-Detection Methods

These types of methods perform the tracking and detection processes together in a singular framework without a distinct detector. These methods typically require manual initialization, and the number of objects in the initial frame is fixed. Subsequently, these objects are located and tracked throughout the succeeding frames. We review these methods in the subsections below.

#### 2.1.1. Filter-Based Methods

The methods under this category primarily employ Kalman and Particle filters for the tracking process. For instance, in Huang et al. [17], an adaptive particle filter coupled with an effective proposal distribution was proposed to handle cluttered backgrounds and occlusions efficiently. To facilitate a more diverse proposal distribution, an adaptive motion model was used. Additionally, the template correlation was integrated with motion continuity and trajectory smoothness in the observation likelihood, further eliminating visual distractions.

In the study by Habibi et al. [18], tracking was integrated with a super-resolution technique wherein a high-resolution image was created from multiple low-resolution images. Given that super-resolution enhanced the visual quality of small objects, the process provided more tracking information, thereby increasing precision. The tracking process was then conducted through an adaptive Particle filter, as proposed in Huang et al. [17].

Liu et al. [19] put forth an approach grounded in super-resolution, using convolutional neural network (CNN) to track small objects. A deep-learning network was deployed to enhance the visual quality of small objects, subsequently improving the tracking performance. A Particle filter was then employed for tracking [20].

In their research, Wu et al. [21] proposed an enhanced kernel correlation filter (KCF)-based approach for tracking small objects in satellite videos. Occlusion presents a significant hurdle in object tracking, especially apparent in satellite videos due to the minute size of objects, making them more susceptible to occlusion. The methodology in this study used the average peak correlation energy and the peak value of the response map to determine potential object occlusion. The object’s subsequent location was forecasted employing a Kalman filter.

#### 2.1.2. Search-Based Methods

Search-based methods attempt to discover the best tracks through extensive searching, with a particular focus on small object tracking. Notable among these methods is the algorithm proposed by Blostein et al. [22], dubbed Multiple Hypothesis Testing (MHT). This method operates under the assumption that the intensity values of background and noise are lower than the mean target intensity. In this approach, the track tree roots are chosen from a predetermined number of points with the highest intensity value. For each root, the algorithm selects neighboring points in the subsequent frame to construct a track tree. Within MHT, there are two thresholds—T_1 and T_2—against which each point on the track is compared. If the new point surpasses T_2, the algorithm records the track and proceeds to the next frame. If the point falls below T_1, the track is rejected. However, if the new point lies between T_1 and T_2, the algorithm defers the decision to the next frame. Ultimately, the tree is pruned to yield a desired number of tracks. Nonetheless, this method faces challenges when tracking fast-moving small objects, as the search area increases exponentially. This makes current MHT algorithms computationally impractical for objects moving at speeds exceeding 1 pixel/frame. In response to this problem, Ahmadi et al. [23] utilized the Multi-Objective Particle Swarm Optimization algorithm (MOPSO) [23] to identify the most optimal track within each root.

Salari et al. [24] presented an effective algorithm for tracking dim targets within digital image sequences. The algorithm operates in two stages: noise removal and tracking. Initially, the Total Variation (TV) filtering technique is employed to improve the Signal Noise Ratio (SNR) and eliminate the image’s noise. Subsequently, to detect and track dim tiny targets, a genetic algorithm with associated genetic operators and encoding is used. In the study by Shaik et al. [25], Bayesian techniques were deployed for the detection and tracking of targets in infrared (IR) images. The algorithm begins by applying preprocessing to incoming IR targets to reduce noise and segmentation. The initial position of the object is ascertained utilizing ground truth (GT) data. Subsequently, a grid composed of segments around the target’s position in the ensuing frame is chosen, and regions with high-intensity within this segment are highlighted. Employing Bayesian probabilistic methodologies, the likelihood of the object shifting its position from the current frame to any high-intensity location within this grid is then calculated. The position suggesting the highest probability is chosen, and the object’s position in the following frame is established. Given that an object’s intensity may not necessarily be the highest in a frame, the position and intensity of the object in the previous frame are considered in the Bayesian probabilistic equation to determine its position in the next frame.

### 2.2. Track-by-Detection Methods

In track-by-detection methods, the detector autonomously identifies the desired object or objects in each frame. Subsequently, the tracking process is conducted to associate the detected objects across successive frames. The objects are initially detected, and then their trajectories are linked. This category of methods is capable of handling frames containing a variable number of objects. Thus, if a new object enters the scene, unlike methods in the previous category, these methods will not face any complications. This flexibility has made them more popular, and the majority of studies presented in the field of small object detection and tracking fall within this category. However, it should be noted that the efficacy of these methods largely depends on the accuracy of the detector they utilize. In the following sections, we will investigate and review such methods.

#### 2.2.1. Background Detection-Based Methods

Methods utilizing frame differencing and background subtraction gained considerable attention due to their simplicity and robust performance in real-time applications. For instance, the study outlined by Archana et al. [26] employed this technique to detect and track a tennis ball and players within video frames. Initially, the input images are smoothed, and background images are accumulated to create an average background model. To execute detection in the current frame, the difference between the frame and its predecessor is calculated, and the logical AND operation is executed between the difference image and the derived background. Subsequently, the ball and player candidates are determined based on the size of the identified contour. Ultimately, tracking is executed separately for the ball and players using the centroid of the detected contour. In another study, [27], the authors proposed an adaptive background subtraction algorithm for the detection and tracking of moving objects within a video sequence. They extracted the video’s background image and applied a median filter to denoise the video sequence. The objects were then identified utilizing an adaptive background subtraction algorithm along with mathematical morphology. The tracking process employed the objects’ centers. Importantly, this method incorporates an update of the background at each stage.

An alternative methodology was introduced by Srivastav et al. [28], which incorporated three-frame differencing and background subtraction for detecting moving objects in videos. The procedure commences with the selection of three successive frames from the image sequence. Subsequently, the difference between the first and second frames is computed, denoted as D_1. Similarly, the outcome of the difference between the second and third frames is labeled as D_2. If DB signifies the result of subtracting the background from the current frame, moving objects are detected by implementing a pixel-wise logical OR operation on D_1, D_2, and DB. Finally, background noise is eliminated by utilizing a median filter.

Zhu et al. [29] incorporated three-frame differencing and operations such as “AND” and “XOR” for swift detection of moving objects. The difference image, p_1, is obtained by calculating the difference between the initial two frames, and p_2 is obtained from the difference between the second and third frames. Subsequently, a new image, p_3, is created by performing p_1 AND p_2. The next step involves obtaining p_2 XOR p_3, resulting in a new image, p_4. Ultimately, the detection image is derived from p_1 AND p_4. Following detection, noise is mitigated using post-processing algorithms. In their research, Yin et al. [30] proposed an algorithm known as Motion Modeling Baseline (MMB), designed to detect and track small, densely clustered moving objects in satellite videos. The process commences with the extraction of candidate slow-moving pixels and region of interest proposals using accumulative multi-frame differencing (AMFD). The full targets are then efficiently detected using low-rank matrix completion (LRMC). Lastly, the motion trajectory-based false alarm filter mitigates false alarms by compiling the trajectory over time, underlining that authentic moving targets are more likely to exhibit continuous trajectories.

Zhou et al. [31] presented a study that utilized an efficient and unsupervised approach, employing background subtraction for object delineation in Wide Area Motion Imagery (WAMI). Initially, background subtraction is used to detect low contrast and small objects, leading to the extraction of objects of interest. Following this, a convolutional neural network (CNN) is trained to reduce false alarms by considering both temporal and spatial data. Another CNN is subsequently trained to forecast the positions of several moving targets within a specified area, thus reducing the complexity of the necessary multi-target tracker. A Gaussian Mixture-Probability Hypothesis Density (GM-PHD) filter is finally employed to correlate detections over time.

Teutsch et al. [32], proposed an algorithm for detecting moving vehicles in Wide Area Motion Imagery that enhanced object detection by utilizing two-frame differencing along with a model of the vehicle’s appearance. The algorithm amalgamates robust vehicle detection with the management of splitting and merging, and applies an appearance-based similarity measure to estimate assignment likelihoods among object hypotheses in consecutive frames.

Aguilar et al. [33] proposed a multi-object tracking (MOT) technique for tracking small moving objects in satellite videos. They used a patch-based CNN object detector with a three-frame difference algorithm to concentrate on specific regions and detect adjacent small targets. To improve object location accuracy, they applied the Faster Region-based convolutional neural network (Faster R-CNN) [34] since the three-frame difference algorithm neither regularizes targets by area nor captures slow-moving targets. Furthermore, they applied a direct MOT data-association approach facilitated by an improved GM-PHD filter for multi-target tracking.

This approach was advanced by Aguilar et al. [35], where the performance of Faster R-CNN’s object detection was significantly boosted by merging motion and appearance data on extracted patches. The new approach comprises two steps: initially obtaining rough target locations using a lightweight motion detection operator and, then, to enhance the detection results, combining this information with a CNN. An online track-by-detection methodology is also applied during the tracking process to convert detections into tracks based on the Probability Hypothesis Density (PHD) filter.

In the research conducted by Lyu et al. [36], a real-time tracking algorithm was introduced, specifically designed for ball-shaped, fast-moving objects, leveraging frame difference and multi-feature fusion. The process initiates by applying frame difference between two consecutive frames, after which the resulting differential image is segmented into smaller contours. A multi-feature-based algorithm is then used to determine if these are moving areas with ball-shaped objects.

Hongshan et al. [37] proposed a wiener filter-based infrared tiny object detection and tracking technique that optimizes filtering under stable conditions based on the least mean square error metrics. Given that the background is distributed in the image’s low-frequency part and the high-frequency part primarily encompasses small objects, an adaptive background suppression algorithm is performed, taking advantage of the low-pass Wiener filter’s characteristics. Appropriate segmentation then reveals potential targets. The relationship between multiple frames, including the continuity and regularity of target motion, is utilized for detection and tracking.

In the research conducted by Deshpande et al. [38], they applied max-mean and max-median filters on a series of infrared images for the detection of small objects. The initial step involves applying either the max-mean or max-median filter to the unprocessed image. Subsequently, the filtered image is subtracted from the original one to highlight potential targets. A thresholding step, which is guided by the image’s statistical characteristics, limits the quantity of potential target pixels. Finally, the output images are cumulatively processed to track the target. The post-processing algorithm is equipped to detect the continuous trajectory of the moving target.

#### 2.2.2. Classical Computer-Vision-Based Methods

These types of methodologies use algorithms and techniques that are based on mathematical models and heuristics to execute detection and tracking processes. For example, a study conducted in 2016 used frequency and spatial domain information to track small dim objects in infrared image sequences [39]. This method consists of three stages: initially, each frame produces six high-frequency sub-bands using the Dual-Tree Complex Wavelet Transform (DT-CWT). The potential targets in these high-frequency sub-bands are then detected by the Constant False Alarm Rate (CFAR) detection module. Finally, the potential targets are refined using an SVM classifier.

In their research, Ahmadi et al. [40] proposed an algorithm to identify and monitor small and dim infrared targets by integrating three mechanisms of the Human Visual System (HVS). The procedure involves four stages. Initially, a multi-scale Difference of Gaussians (DOG) filter is applied to the current image frame to create a series of feature maps at different scales. These maps are subsequently consolidated to form a saliency map. In the next stage, a Gaussian window is established at a location near the target, referred to as the visual attention point, on the saliency map. After normalizing the entire image, the target’s position within the current frame is determined. Finally, the Proportional-Integral-Derivative (PID) algorithm is used to forecast the location of the visual attention point in the following frame.

Dong et al. [41] also introduced a method for detecting small moving targets in infrared images. Initially, the points of interest, including moving targets, are extracted using DOG filters. These interest points are tracked across multiple frames, and the relationship between these points in the first and last frames is established. Ultimately, based on the relationships, these interest points are divided into two clusters: target points and background points.

In the study by Zhang et al. [42], an algorithm was presented to detect and track dim moving points with low Signal-to-Noise Ratio (SNR) in IR image sequences. This algorithm first applies a temperature non-linear elimination and Top-hat operator to preprocess the original images. Then, a composite frame is obtained by reducing the three-dimensional (3D) spatio-temporal scanning of an object to 2D spatial hunting. The tracking process, conducted in the final step, finds the object trajectory using the condition of a constant false alarm probability.

Lastly, Shaik et al. [43] proposed a strategy based on object preprocessing, tracking, and classification for infrared target sequences. Initially, preprocessing stages comprising normalization, rank order filtering to enhance the object features in the frame, and morphological operations to remove noise from frames are conducted in sequence. For tracking multiple objects, a correlation matrix is utilized, and the objects’ positions in the frames are determined. Finally, a self-organizing map (SOM) is employed for classification based on various features, such as statistics and the object’s shape.

An aggregation signature was utilized by Liu et al. [44] for small object tracking. This method suppresses the background through the aggregation signature, achieving highly distinctive features for small objects. The approach also generates a saliency map for frames and performs tracking. The tracker employs both the target’s prior information and the context data to relocate the tracked target.

In the study by Tzannes et al. [45], temporal filters were applied to detect point targets in IR imagery. The central concept involves exploiting the difference in temporal profiles between target and clutter pixels. The “temporal profile” refers to the changing pixel value over a short period in an IR sequence. The pixels of a point target exhibit a pulse-like temporal profile. In this study, the implementation of the continuous wavelet transform (CWT) was explored for these temporal pixel profiles.

Bae et al. [46] introduced a technique for detecting small targets in sequences of infrared images, grounded in the cross-product of temporal pixels, relying on temporal profiles. Small targets display temporal characteristics that are distinguishable from various backgrounds. Consequently, the approach discriminates target and background pixels based on the cross-product of pixel values within the temporal profile. The method aggregates temporal background pixels to forecast the temporal background in the target region. Small target detection is then executed by measuring the absolute difference between the original temporal profile and the predicted background temporal profile, postulating that the absolute difference in target pixels exhibits a higher grey level. Ultimately, the target’s trajectory is determined using a threshold that validates the presence or absence of a target based on each pixel’s value in the calculated difference.

Bae [47] proposed a spatial and temporal bilateral filter (BF) intended for the identification of small trajectories in his study. This approach functions through the extraction of spatial target details via a spatial BF, and temporal target data via a temporal BF. The detection of small targets is realized by subtracting the anticipated spatial and temporal background profiles from the original infrared image and the original temporal profile, respectively. Similar to previous methods, this technique employs a threshold to ascertain a small target trajectory.

In the work presented by Choudhary [48], an automated process for the identification and tracking of targets within sequences of infrared images was achieved through the application of morphological connected operators. It involved two stages: intraframe and interframe. In the intraframe stage, the background is minimized to enhance the targets’ visibility, and a binary image is created using adaptive double thresholding. In the interframe stage, the algorithm first assigns labels to the binary detections from the previous stage, ensuring that detections associated with the same target receive the same label. Finally, targets not consistently detected in the sequence are eliminated.

In the research conducted by Son et al. [49], a model for tracking minuscule drones was put forth, featuring a predictor based on the Kalman filter and multiple trackers. This model is composed of two distinct trackers, a predictor, and a refining operation. The first tracker employs motion flow to identify a moving target, while the second utilizes histogram features to establish the region of interest. The trajectory of the target is then forecasted using the Kalman filter. Lastly, the refinement operation is employed to settle on the precise location of the target.

#### 2.2.3. Deep-Learning-Based Methods

In this category, deep learning is used to detect small objects and, afterwards, their tracking. Deep networks can learn features and classifiers for detection and tracking tasks. For example, convolutional neural networks (CNNs) can learn intricate and distinctive features from massive datasets, deal with various kinds of objects and scenes, adjust to changing situations and appearance changes, and transfer to new settings and domains. They have several benefits, such as high precision, reliability, and flexibility. However, CNN-based methods also have some limitations, such as high computational expense, large memory usage, vulnerability to scale and occlusion, and challenge in handling small objects [35].

In the study conducted by Zhang et al. [50], three conventional convolutional neural networks, namely Faster R-CNN, YOLOv3 [51], and YOLOv3 tiny, were employed for the real-time detection and tracking of a golf ball within video sequences. Adiscrete Kalman filter was utilized during the tracking phase, predicting the golf ball’s position based on previous observations. To improve accuracy and processing speed, image patches, rather than full images, were employed for detection purposes. Aktaş et al. [52] employed aerial imaging for small object detection and tracking. Given the unique challenges of aerial imagery due to wide-field images and tiny target objects, the standard Faster R-CNN model is modified. The goal is to use both spatial and temporal information from the image sequence, as appearance information alone is insufficient. The method adjusts the anchors in the Region Proposal Network (RPN) stage and optimizes the intersection over union (IoU) for small objects. After improving detection performance, the Deep SORT algorithm [53] is applied for small object tracking.

Behrendt et al. [54] introduced a system to detect, track, and perform 3D localization of traffic lights for automated vehicles using deep learning. A trained neural network detects traffic lights (as small as 3 × 10 pixels), while the tracker triangulates the traffic lights’ locations in the 3D space using stereo imagery and vehicle odometry information. A neural network is then used to correct the location estimate. In the study by Hurault et al. [55], a self-supervised soccer player detector and tracker robust to small players and suitable for wide-angle video games was presented. The detection framework involves sequential training of a teacher and student for domain adaption and knowledge distillation, followed by player trajectory tracking using spatial and visual consistency. Zhu et al. [56] proposed a Multilevel Knowledge Distillation Network (MKDNet), which enhances feature representation, localization abilities, and discrimination for tiny object tracking. MKDNet performs three levels of knowledge distillation: score, feature, and IoU-level distillation. In the study by Liu et al. [57], transformers were employed for aerial small object tracking. Transformer, a popular network structure in deep learning, uses the attention module to provide a global response between the template frame and the search frame, effectively supplementing contextual information for small objects. Therefore, it can address the issue of poor feature expression capability in small objects. Also, a template update strategy is applied to manage template updates under occlusion, out-of-view, and drift conditions.

Huang et al. [58] proposed a deep autoencoder architecture called TrackNet for tracking tennis balls from broadcast videos. TrackNet generates a detection heatmap from either a single frame or several consecutive frames to locate the ball and learn flying patterns. Yoshihashi et al. [59] presented the Recurrent Correlational Network (RCN) for joint detection and tracking of small flying objects, where detection and tracking are jointly carried out on a multi-frame representation learned through an end-to-end, trainable, and single network. A convolutional long short-term memory network learns informative appearance change in the detection process, enabling correlation-based tracking over its output. Another approach was proposed by Marvasti-Zadeh et al. [60], which used a two-stream multitask network and an offline proposal approach to track aerial small objects. The network, aided by the proposed proposal generation strategy, leverages context information to learn a generalized target model and handle viewpoint changes and occlusions. The reviewed methods are subsequently categorized based on the above taxonomy in Table 1.

## 3. Types of Public Data Sets Used for Small Object Detection and Tracking

The datasets used for small object detection and tracking are essential for evaluating and benchmarking the performance of various algorithms in this field. These datasets can be broadly classified into two groups: spectrum-based video datasets and source position-based video datasets. Spectrum-based datasets include infrared (IR) videos whereas source position-based datasets include aerial videos, satellites videos, and normal videos. Note that videos captured by satellites or Unmanned Aerial Vehicles (UAVs) present more challenges due to larger dimensions, smaller objects, and the presence of many more objects compared to normal videos. The difficulty is especially pronounced in satellite videos due to the higher altitude of the target object. The following are some popular and publicly available datasets in the field.

### 3.1. Small90 and Small112

Small90 is one of the most comprehensive datasets in the field of small object tracking in normal videos. It includes 90 sequences of annotated small-sized objects. The dataset was created from existing tracking datasets by selecting videos with an object area size ratio smaller than 0.01 of the entire image. Additional challenges, such as target drifting and low resolution, were also considered. Small112 extends Small90 by adding 22 more challenging sequences. Each sequence in these datasets is classified using 11 attributes for a comprehensive study of tracking approaches [44].

### 3.2. Large-Scale Tiny Object Tracking (LaTOT)

LaTOT stands for Large-scale Tracking of Tiny Objects, and it was developed as a response to the limitations observed in the Small90 dataset, particularly its small-scale size and limited challenges. The LaTOT dataset consists of 434 video sequences, encompassing more than 217,000 frames, all captured in real-world scenarios. Each frame in the dataset is meticulously annotated with high-quality bounding boxes. Additionally, the dataset incorporates 12 challenging tracking attributes aimed at encompassing a wide range of viewpoints and scene complexities [56].

### 3.3. Video Satellite Objects (VISO)

VISO is a large-scale dataset designed for the purpose of detecting and tracking moving objects in satellite videos. It comprises 47 high-quality satellite videos, which were acquired using Jilin-1 satellite platforms. Within this dataset, there are 3711 object tracking trajectories, and it contains over 1.6 million instances of interest specifically for object detection tasks. Each image in the dataset possesses a resolution of 5000 × 12,000 pixels, and it encompasses a diverse array of objects varying in scale. The dataset encompasses various types of moving objects, including trains, cars, ships, and planes, and it is formulated to incorporate seven key challenges commonly encountered in object tracking research [30].

### 3.4. UAV123

UAV123 is a dataset for object tracking in UAV aerial imagery, comprising over 110K frames and 123 video sequences. The sequences are high-resolution, fully annotated, and captured from an aerial perspective at low altitudes. The dataset has three sections: high-quality sequences taken at diverse heights, noisy and lower-quality sequences, and synthetic sequences [61].

### 3.5. Dataset of Object deTection in Aerial Images (DOTA)

DOTA, which stands for detection in aerial images, is a valuable resource that facilitates object detection tasks in Earth Vision. This dataset contains a substantial collection of 1,793,658 instances distributed across 11,268 images, encompassing 18 commonly encountered categories. Each object within the dataset is annotated with a bounding box, which may be either horizontal or oriented. Despite the dataset containing a significant number of small objects, it was observed that these objects are predominantly concentrated in a limited number of categories, specifically within the “small-vehicle” category. This concentration can be attributed to the high diversity of orientations observed in overhead view images and the substantial variations in large-scale amongst the instances [62].

### 3.6. Vision Drone (VisDrone)

VisDrone is an extensive dataset collected through the use of drones, spanning various urban and suburban regions across 14 distinct cities in China. This dataset is curated to facilitate four essential computer vision tasks, namely image object detection, video object detection, single object tracking, and multi-object tracking. For the image object detection track, VisDrone consists of 10,209 images, each having a resolution of 2000 × 1500 pixels, and the dataset encompasses a total of 542,000 instances representing 10 typical object types commonly found in traffic scenes. The imagery in VisDrone was captured using drones from multiple viewpoints within diverse urban settings, leading to variations in viewpoints and significant occlusion. Consequently, the dataset includes a substantial number of small objects [63].

### 3.7. Tsinghua-Tencent 100K (TT100K)

The TT100K dataset is utilized for realistic traffic sign detection in normal videos, encompassing 30,000 instances of traffic signs distributed across 100,000 images. These images represent 45 distinct classes of typical Chinese traffic signs. Each traffic sign within the TT100K dataset is meticulously annotated, providing detailed bounding boxes and instance-level masks. The images in TT100K were captured using Tencent Street Views, which offers a wide range of weather conditions and illumination settings, adding to the dataset’s realism. Notably, TT100K exhibits a considerable number of small instances, leading to a long-tail distribution, with approximately 80% of instances occupying less than 0.1% of the entire image area [64].

## 4. Performance Evaluation Metrics

In the realm of small object detection and tracking, the majority of methods employ a track-by-detection approach, commonly evaluating their detection and tracking modules individually. This section elaborates on several evaluation metrics utilized in the quantitative appraisal of algorithms during these two stages. During the detection stage, primary metrics encompass Precision, Recall, F1-score, the Precision-Recall (PR) curve, Average Precision (AP), and mean Average Precision (mAP). Regarding the tracking stage, evaluation measures encompass Overlap Success Rate (OSR), Distance Precision Rate (DPR), Multiple Object Tracking Accuracy (MOTA), Multiple Object Tracking Precision (MOTP), and Completeness.

### 4.1. Metrics for Detection

- Precision and Recall:

Before introducing these metrics, it is necessary to explain the following concepts:

– True Positive (TP): A ground-truth bounding box that is detected correctly;
– False Positive (FP): Mistakenly detecting an object that does not exist or detecting an object that does exist in the wrong place;
– False Negative (FN): This term refers to an instance where a ground-truth bounding box goes undetected.

The ability to distinguish between True Positive (TP) and False Positive (FP) is critical for object detection [65]. Additionally, False Negative (FN) is the name given to missed true targets. Precision is defined as the ratio of true positives to the detected targets as follows:

(1) $$Precision=\frac{TP}{TP+FP}$$

A detector’s capability to capture the targets is calculated using Recall [65]. This metric is defined as the ratio of TP to the number of all existing true targets:

(2) $$Recall=\frac{TP}{TP+FN}$$

- F1-score

A traditional criterion for categorizing objects into either being targets or not is the F1-score that is equivalent to Precision and Recall’s harmonic mean [30,35,66], i.e.,

(3) $$F1-score=\frac{2\times Recall\times Precision}{Recall+Precision}$$

Despite the widespread usage of Precision, Recall, and F1-score as evaluative measures for object detection algorithms, these metrics have inherent limitations. It is crucial to acknowledge their potential for unfairness before application and to discern chance or base case levels of the statistic. In certain cases, a system with objectively lower quality, in terms of informedness, may appear to possess superior quality when judged using these popular metrics [67].

- Precision-Recall (PR) curve

The Precision-Recall (PR) curve serves as a graphical representation depicting the trade-off between precision and recall at different threshold levels. A high precision value indicates a low false-positive rate, whereas high recall corresponds to a low false-negative rate. A large area under the PR curve signifies both high recall and precision, reflecting accurate classifier results (high precision) and a substantial proportion of true positives (high recall) [30]. It is important to note that the shape of this curve is influenced by the threshold chosen, which, in turn, impacts the trade-off between precision and recall.

- Average-Precision (AP)

The AP metric, which is defined as the area under the Precision-Recall curve, is a widely accepted measure of detection model accuracy [65]. However, calculating an accurate Area Under the Curve (AUC) can be challenging in practice, primarily due to the characteristic zigzag shape of the precision-recall plot. To mitigate this issue, the zigzag behavior is eliminated by processing the precision-recall curve prior to estimating the AUC. The two most common techniques for this processing are the 11-point interpolation and all-point interpolation. The 11-point interpolation method aims to capture the essence of the precision-recall curve’s shape by calculating the average maximum precision values at a series of 11 evenly spaced recall levels [0, 0.1, 0.2, ..., 1]. The shape of the precision × recall curve is summarized by the 11-point interpolation as:

(4) $${AP}_{11}=\frac{1}{11}\sum_{R\in \left\{0,0.1,\ldots ,0.9,1\right\}}P_{interp}(R)$$

(5) $$P_{interp}(R)=\underset{\tilde{R}:\tilde{R}\geq R}{\max}P(\tilde{R})$$

Rather than using the observed precision P® at each recall level R, as specified in this definition, one can determine the Average Precision (AP) by considering the maximum precision $P_{interp}(R)$ with a recall value equal to or greater than R.

In the all-points interpolation method, instead of interpolating only at 11 evenly spaced points, one can choose to interpolate at all points as follows:

(6) $${AP}_{all}=\sum_{n}\left(R_{n+1}-R_{n}\right)P_{interp}(R_{n+1})$$

(7) $$P_{interp}(R_{n+1})=\underset{\tilde{R}:\tilde{R}\geq R_{n+1}}{\max}P(\tilde{R})$$

In this case, the maximum precision with a recall value equal to or greater than $R_{n+1}$ is used to interpolate the precision at each level. This provides a more accurate representation of AP than merely relying on the precision observed at a limited number of points. Average precision has some limitations. First, it assumes that precision is a continuous function of recall, which is not true in practice. Moreover, this measure ignores the size and shape of the objects, and it requires binarizing the output for multi-class or multi-label classification.

- mean Average-Precision (mAP)

Within a specific dataset, the accuracy of object detectors is evaluated across all classes using the mAP, which is simply the average of the AP scores across all classes as follows:

(8) $$mAP=\frac{1}{N}\sum_{i=1}^{N}{AP}_{i}$$

where *N* indicates how many classes are being evaluated in total, and ${AP}_{i}$ is the AP for the *i*th class [65].

### 4.2. Metrics for Tracking

Evaluation criteria for the tracking stage are divided into two categories: single object tracking and multiple object tracking, detailed as follows.

#### 4.2.1. Single Object Tracking

Key metrics employed within this category involve the Overlap Success Rate (OSR) and the Distance Precision Rate (DPR). The Distance Precision Rate (DPR) is determined by computing the ratio of frames where the center location error, denoted as the Euclidean distance between the centers of the predicted box (B_p_) and the ground truth box (B_g_), falls below a designated threshold α. On the other hand, the Overlap Success Rate (OSR) is defined as the proportion of frames where the overlap ratios with the ground-truth box surpass a specified threshold β. The overlap ratio is mathematically expressed in Equation (9):

(9) $$S=\frac{\left|B_{p}\cap B_{g}\right|}{\left|B_{p}\cup B_{g}\right|}$$

where $\left|.\right|$ indicates the number of pixels in an area ∩ and ∪ show the intersection and union of two areas, respectively [68].

OSR and DPR rely on the selection of thresholds for determining the overlap rate and the distance mistake, which can influence the sensitivity and specificity of the evaluation. Furthermore, these measures neglect the scale variation of the target, which is a frequent difficulty in tracking [69]. These are two important limitations for these metrics.

#### 4.2.2. Multiple Object Tracking

- Multiple Object Tracking Accuracy (MOTA)

MOTA is a widely used metric for assessing the overall performance of a multiple-object tracker. It evaluates the quality of the recovered tracks by considering identity switches, missed targets, and false positives as follows:

(10) $$MOTA=1-\frac{\sum_{t}\left(m_{t}+{fp}_{t}+{mme}_{t}\right)}{\sum_{t}g_{t}}$$

Here, $g_{t}$ represents the number of ground-truth objects at frame index t. The terms $m_{t}$, ${fp}_{t}$, and ${mme}_{t}$ denote missed targets, false positives, and ID switches at the t-th frame, respectively. MOTA provides an aggregate count of tracking errors and ranges from (−∞,1] with negative values indicating poor performance and one representing the highest possible score [70].

- Multiple Object Tracking Precision (MOTP)

MOTP considers the distance between the detected objects and the ground truth objects. It is a representative measure of a tracker’s precision performance and is defined by the following equation:

(11) $$MOTP=\frac{\sum_{t,i}d_{t}^{i}}{\sum_{t}c_{t}}$$

where $d_{t}^{i}$ is the distance between the object $o_{i}$ and its corresponding hypothesis and $c_{t}$ is the total number of matches in frame *t* among the true targets and the hypothesized objects. The MOTP score falls in the interval [0,∞), where a score of 0 indicates good performance, and higher values indicate poorer performance [71].

It should be noted that MOTA and MOTP are not appropriate for real-time tracking applications, because they need the ground truth information of all the frames in a video sequence, which is not accessible in real-time situations. Moreover, they are not adaptable to multi-class tracking problems, because they suppose that all the objects are in the same class and have the same appearance model [72].

- Completeness

Completeness metrics evaluate the overall continuity of the tracking trajectory. Specifically, trajectories resulting from combined tracker outputs can be classified into three categories: Mostly Tracked (MT), Partially Tracked (PT), and Mostly Lost (ML). The MT category suggests that the tracker output covers more than 80% of the ground truth trajectory’s length, whereas the ML category signifies it covers less than 20%. The PT category applies to all other instances. Therefore, an optimal tracker should ideally produce a larger proportion of sequences classified as MT and a smaller proportion as ML [71].

Completeness metrics have some limitations. In this way, they do not measure the accuracy of the location of the tracked objects, which can influence the quality of the tracking results. Furthermore, these criteria do not consider the identity switches of the tracked objects, which can lead to confusion and mistakes [73].

## 5. Challenges and Future Trends

**Figure 2:**
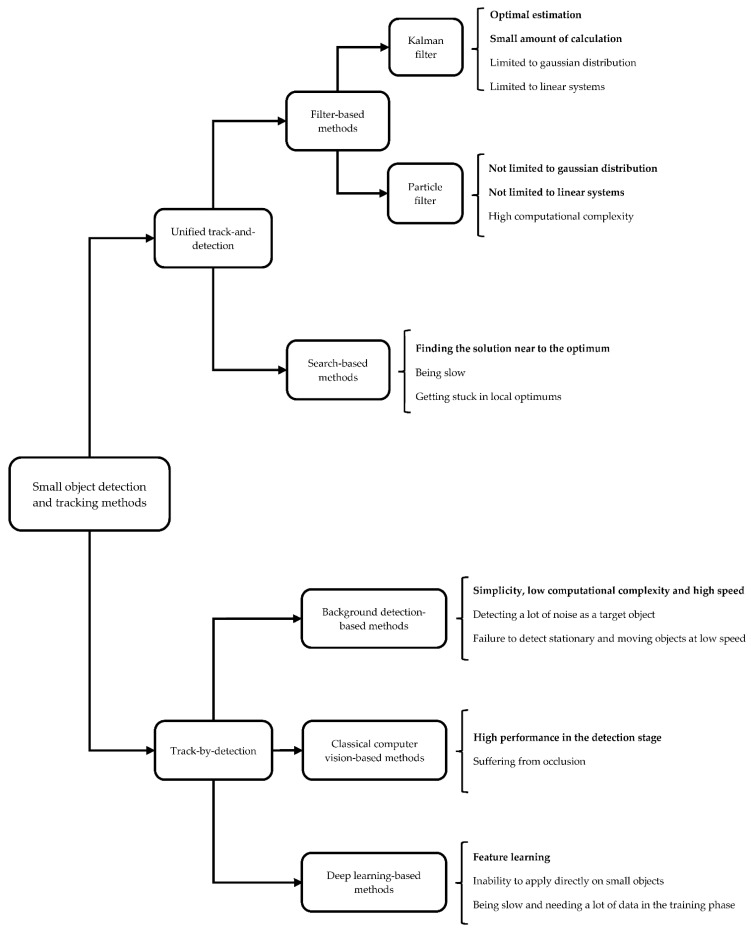
The taxonomy of detection and tracking methods of small objects along with their advantages and drawbacks.

In this section, we initially contrast the methods reviewed, focusing on their respective strengths and shortcomings, before delving into a detailed analysis. We also discuss the challenges faced by existing object detection and tracking methods and explore potential future trends to overcome these limitations. We also introduce a sensor fusion-based approach that incorporates 3D LiDAR into the object detection and tracking processes. Table 2 compares unified track-and-detection methods with tracking-by-detection methods based on their advantages and shortcomings. Additionally, Figure 2 individually delineates the taxonomy of small object detection and tracking methods along with the merits and pitfalls of existing methods across each category. Note that the merits of methods across each category are highlighted in boldface style.

According to Table 2, it can be concluded that the advantage of unified track-and-detection methods is that these types of methods perform detection and tracking processes in a unified manner and without a detector. In other words, these methods are free of object detectors. However, their shortcomings are that they require a manual initialization with a fixed number of objects in the first frame such that these objects are located and tracked in the next frames. This means that when new objects enter the scene, these methods are not able to manage them and so have limited applications. On the other hand, track-by-detection methods are more popular and have more applications due to performing a detection stage and automatically detecting objects in each frame by detection algorithms. The advantages of these methods are automatic initialization and managing the scene with a varying number of objects due to the detection stage. However, the performance of these methods is highly dependent on the performance of the utilized object detector which is their main shortcoming. This means that poor performance in the detection process can adversely affect the performance of the tracking process. In brief, tracking-by-detection methods are more widely used within the field of small object detection and tracking because of discovering new objects and terminating disappearing objects automatically. That is while unified track-and-detection methods cannot deal with these challenges.

As Figure 2 elucidates, in the context of Filter-based methods, the Kalman filter generally enhances the tracking process’s efficiency through optimal estimations. Its simplicity and minimal computational requirements make it apt for tasks necessitating high real-time responsiveness. Nonetheless, the Kalman filter’s assumptions of Gaussian distribution for state variables and its suitability only for linear systems are limitations. The particle filter resolves these constraints, but its elevated computational cost renders it unsuitable for real-time applications. It should be mentioned that there are a lot of Kalman filter variations, such as the Extended Kalman Filter (EKF) and Unscented Kalman Filter (UKF), which are the famous heuristic variations of the Kalman filter for nonlinear systems with Gaussian distribution [74,75]. Search-based methods, on the other hand, suffer from slow speeds, high computational complexity due to the search process, and also the potential to become trapped in local optima. Hence, they are unsuitable for real-time applications. Yet, these methods’ utility lies in their ability to deliver near-optimal solutions with acceptable error margins.

Background-based methods boast simplicity, low computational complexity, and high speed. As a result, these methods have good performance in real-time applications. However, they tend to generate considerable noise as the target object, necessitating post-processing methods for noise removal. Moreover, they might fail to detect certain objects, particularly those that are stationary or moving slowly. Classical computer-vision-based methods exhibit excellent performance in object detection, effectively identifying objects within video frames using mathematical and heuristics techniques. Unfortunately, these methods often overlook the challenge of occlusion during the tracking stage, a common and critical issue in object tracking. This occurs when objects appear to blend or merge due to close proximity or background integration, potentially causing the tracker to lose or erroneously track objects after overlapping [76]. In other words, occlusion means that an object is partially or completely hidden by other objects, which reduces the appearance and location information of the object. This issue is particularly prevalent with small objects due to their diminutive size.

To counter occlusion, trackers typically employ data association. However, there are various methods for small object detection and tracking under occlusion that can modify accuracy, speed, robustness, and generality. Some of these methods are: using a large number of anchor points to cover a wider search space, using multiple-frame receptive networks for small object tracking, using motion as a strong cue for small object detection in videos, using correlation filter to maintain the identity and trajectory of objects over time, and using reconstruction methods to recover the appearance and location of the object after occlusion [77,78]. For instance, Wang et al. [79] addressed occlusion by calculating the correlation between different frames and learning motion information. In the study by Wojke et al. [53], the Deep SORT algorithm integrated motion and appearance information to solve this issue by utilizing Mahalanobis distance and a convolutional neural network (CNN) trained on a large-scale person re-identification dataset. It should be noted that the real-time performance and computational complexity of classical computer-vision-based methods depend on several factors, such as the size and quality of the input images, and the type and complexity of that methods.

Deep-learning-based methods offer the advantage of feature learning, facilitating end-to-end functionality. However, these methods face challenges when directly applied to small objects as these possess limited and poor features, which may get lost during deep network processing, because these networks have many layers and, as a result, include many processes. This challenge means that small objects have fewer and less distinguishable features than larger objects due to their small shape size. This makes it difficult for deep networks to learn and extract suitable features for small object detection and tracking. To solve this problem, various methods have been proposed to enhance and improve the feature information of small objects that can improve accuracy, speed, robustness, and generality. Some of these methods are: using feature enhancement modules (FEM) that can use feature aggregation structures (FAS) and attention generation structures (AGS) to reduce the interference of background by extracting multi-scale contextual information and combining a coordinate attention mechanism, thus improving the perception of small objects, using generative adversarial networks (GAN) to generate new high-quality images that contain small objects, using self-supervised methods to learn features using motion, and using supervised methods to learn features using annotations [80,81].

On the other hand, deep networks require substantial data for the training phase to circumvent overfitting. The cause is that these networks need to learn intricate and distinctive features from massive data, which are normally not accessible for small objects. In addition, small objects usually have a lot of variety and change in their appearance, shape, pose, colour, orientation, and location, which require very diverse and balanced data [82]. Generally, deep networks are slow but there are different methods for small object detection and tracking based on deep learning. As a result, each method has its own advantages and limitations in terms of real-time performance and computational complexity. Hence, it is impossible to find one optimal method for small object detection and tracking that can achieve both high real-time performance and low computational complexity. Various methods may work better for different situations and tasks based on the needs and limitations. Some potential directions for future research are creating more efficient network structures, developing more effective loss functions, using more contextual information, and adding more existing knowledge.

Multiple object tracking (MOT) is another vital consideration. Few works address multiple small object tracking, primarily employing GM-PHD filters for this task. In addition to creating sophisticated appearance and/or motion models to address challenges like scale changes, out-of-plane rotations, and illumination variances (central to single object tracking), MOT involves determining the variable number of objects and maintaining their identities [11]. Traditionally, the track-by-detection paradigm has been employed for MOT, leveraging the inherent separation of detection and data association tasks [83]. Thus, the tracking performance is significantly influenced by the detection results [84,85]. The MOT problem can be viewed as a data association task, aiming to connect detections across video sequence frames [86]. In addition to the common challenges encountered in both SOT and MOT, MOT faces unique concerns such as (1) initializing and terminating tracks, (2) managing interactions among multiple objects, (3) dealing with frequent occlusions, and (4) handling similar appearances. A similar appearance complicates the tracking process as the object’s appearance information is insufficient for differentiation. The study by Dicle et al. [87] applied motion dynamics as a cue to distinguish and track multiple objects with similar appearances. This issue is addressed by formulating the problem as a Generalized Linear Assignment (GLA) of tracklets that are dynamically similar, incrementally associated into longer trajectories. 

Scale variation is another important issue in this domain which means that the relative size of objects to the image or camera is changing, which affects the appearance and location information of the object. This can cause algorithms to fail to detect or track the object correctly. To solve this problem, various methods have been proposed for small object detection and tracking under scale variation in which accuracy, speed, robustness, and generality are improved. Some of these methods are: using image pyramid to provide images with different levels of details, using scale filter to estimate the target scale, using multi-scale feature fusion to reduce scale mismatch, and using multiple-frame receptive networks for small object tracking [56,88,89].

### 5.1. Sensor Fusion-Based Approach

Sensor fusion is a popular approach in modern perception systems, where information from multiple sensors is combined to enhance the overall perception and understanding of the environment. In the context of object detection and tracking, integrating 3D LiDAR data with other sensors, such as cameras, can provide more accurate and comprehensive information about the surrounding objects [90,91].

#### 5.1.1. Hydro-3D: Hybrid Object Detection and Tracking for Cooperative Perception Using 3D LiDAR

One noteworthy system that utilizes this sensor fusion-based approach is the “hydro-3D” framework, a hybrid object detection and tracking system for cooperative perception using 3D LiDAR. By leveraging the high-resolution 3D point cloud data provided by LiDAR, hydro-3D improves the accuracy and robustness of object detection and tracking tasks. The 3D information allows the system to handle various challenges, such as occlusion and object detection in complex scenarios [92].

#### 5.1.2. Automated Driving Systems Data Acquisition and Analytics Platform

The development of advanced object detection and tracking systems, like the hydro-3D, is further facilitated by automated driving systems data acquisition and analytics platforms. These platforms provide a large-scale collection of diverse real-world driving data, enabling researchers and developers to train and validate their algorithms using a vast range of scenarios. The availability of such datasets accelerates progress in the field and fosters the creation of more robust and reliable object detection and tracking systems [93].

#### 5.1.3. YOLOv5-Tassel: Detecting Tassels in RGB UAV Imagery with Improved YOLOv5 Based on Transfer Learning

In recent years, transfer learning has become a widely adopted technique in deep learning-based object detection tasks. YOLOv5-Tassel is an example of a transfer learning-based approach specifically designed for detecting tassels in RGB UAV imagery. By leveraging pre-trained models and fine-tuning on tassel-specific datasets, YOLOv5-Tassel demonstrates enhanced performance and efficiency in detecting small and intricate objects, like tassels, in aerial images [94].

#### 5.1.4. Comparison and Future Trends

In contrast to the methods reviewed earlier, the sensor-fusion-based approach using 3D LiDAR, exemplified by hydro-3D, provides a more comprehensive understanding of the environment. By combining 3D LiDAR data with other sensor inputs, like cameras, the system can address challenges related to occlusion, object detection, and tracking in dynamic and complex scenarios.

Furthermore, the use of automated driving systems data acquisition and analytics platforms facilitates the development and testing of advanced object detection and tracking algorithms, enabling researchers to train and validate their models on diverse real-world data.

For deep-learning-based methods, transfer learning, as demonstrated by YOLOv5-Tassel, becomes crucial in improving the performance of object detection on small objects or objects with limited features. By utilizing pre-trained models and fine-tuning on specific datasets, transfer learning allows the network to learn and generalize better, even with limited training data.

Future trends in object detection and tracking may involve even more sophisticated sensor fusion techniques, integrating data from various sensors, such as LiDAR, cameras, radars, and more. Additionally, the continued advancement of deep learning and transfer learning approaches is likely to enhance the accuracy and efficiency of small object detection and tracking tasks.

In conclusion, the field of object detection and tracking is continually evolving, with sensor-fusion-based approaches and transfer learning playing pivotal roles in overcoming challenges and improving performance. Automated driving systems data acquisition platforms contribute significantly to advancing research and development in this domain. As technology continues to progress, we can expect further breakthroughs that will refine and expand the capabilities of object detection and tracking systems, making them more reliable and applicable across various real-world scenarios.

## 6. Conclusions

This paper reviewed different methods for detecting and tracking small objects, which are important aspects of image and video analysis, mainly due to the limited and inferior features of these objects. We introduced two main categories: unified track-and-detection and track-by-detection methods. The former was split into filter-based and search-based methods, while the latter was divided into background-based, classical computer-vision-based, and deep-learning-based methods. This review also categorized public datasets for small object detection and tracking, dividing them into spectrum-based video datasets and source position-based video datasets. This classification helps researchers to do experimental work. Moreover, we explained the usual evaluation metrics for detection, single object tracking, and multiple object tracking, providing a basis for validating and comparing results.

A significant portion of this review was dedicated to discussing the prevalent challenges in small object detection and tracking. These challenges, encompassing issues such as occlusion, scale variation, speed, computational cost, and the application of deep-learning techniques for small objects, show the complexities in this domain. Moreover, sensor-fusion-based approach with 3D LiDAR can help small object detection and tracking tasks owing to providing more accurate and comprehensive information about the surrounding objects. Concurrently, we assessed the strengths and limitations different methods in each category, providing an evaluation of current approaches and their potential suitability for different scenarios. Furthermore, we outlined potential future research directions, emphasizing the need for better performance and accuracy, especially in dealing with multiple object tracking (MOT). Existing works in MOT are sparse, and more research is needed to handle extra tasks, such as changing numbers of objects, keeping their identities, and managing frequent occlusions or similar appearances.

In conclusion, this comprehensive review is a helpful guide for researchers within the field of small object detection and tracking. Research could focus on dealing with multiple object tracking and improving the performance and accuracy of small object detection and tracking. By outlining the current challenges and future trends, this review gives a roadmap to guide future research in this crucial area of image and video analysis.

## Figures and Tables

**Table 1:** Categorization of the applied methods in the literature.

Types of Methods	Category	References
Unified track-and-detection	Filter-based	[17,18,19,21]
	Search-based	[22,23,24,25]
Track-by-detection	Background-detection-based	[26,27,28,29,30,31,32,33,34,35,36,37,38]
	Classical computer-vision-based	[39,40,41,42,43,44,45,46,47,48,49]
	Deep-learning-based	[50,52,54,55,56,57,58,59,60]

**Table 2:** Comparison of unified track-and-detection methods versus track-by-detection methods.

Types of Methods	Advantages	Shortcomings
Unified track-and-detection	Free of object detector	Manual initialization, Fixed number of objects to be tracked
Track-by-detection	Capable of managing a varying number of objects in video, Automatic initialization	Performance depends on object detection

## Data Availability

Data is contained within the article.
